# Biochemical Composition and Related Potential Nutritional and Health Properties of *Sobrassada de Mallorca*

**DOI:** 10.3390/foods13050761

**Published:** 2024-02-29

**Authors:** Sebastià Galmés, Bàrbara Reynés, Alicia Domínguez-Flores, Silvia Terradas, Antonia María Torres, Andreu Palou

**Affiliations:** 1 Nutrigenomics, Biomarkers and Risk Evaluation (NuBE) Group, University of the Balearic Islands, 07122 Palma, Spain; s.galmes@uib.cat (S.G.); aliciadominguezflores@gmail.com (A.D.-F.); andreu.palou@uib.es (A.P.); 2 Health Research Institute of the Balearic Islands (IdISBa), 07120 Palma, Spain; 3 CIBER of Physiopathology of Obesity and Nutrition (CIBEROBN), Instituto de Salud Carlos III, 28029 Madrid, Spain; 4 Consejo Regulador de la IGP “Sobrasada de Mallorca”, 07006 Palma, Spain; sterradas@sobrasadademallorca.org (S.T.); amtorres@sobrasadademallorca.org (A.M.T.)

**Keywords:** *Sobrassada de Mallorca*, vitamin, mineral, health claims, micronutrients

## Abstract

‘*Sobrassada de Mallorca*’ is an EU PGI (Protected Geographical Indication) -qualified traditional food with important historical, social, and gastronomical relevance. However, its nutritional features are poorly characterized. Here, we studied 15 samples of *Sobrassada de Mallorca* (SM) and 9 samples of ‘*Sobrassada de Mallorca de Porc Negre*’ (SMBP), which are the two types of sobrassada that are PGI-protected. Their composition was assessed under the light of the EU Regulation 1924/2006 on nutrition and health claims (NHC) made on food. Results show the notably high energetic density (588 and 561 kcal/100 g for SM and SMBP, respectively) due to the notable fatty acid (FA) content and the relatively high proportion of unsaturated FAs (≈61% of total FAs) is also noted, mainly oleic acid (39.7 and 45.7%). Moreover, analyses showed that 100 g of both types of ‘*Sobrassada de Mallorca*’ present a ‘significant’ content (at least 15% of the established Nutrient Reference Values) of vitamins A (241 and 232 µg), E (2.67 and 2.67 mg), B_3_ (3.50 and 2.43 mg), B_6_ (0.27 and 0.35 mg), B_12_ (0.65 and 0.56 µg), phosphorus (271 and 186 mg), and selenium (17.3 and 16.2 µg) as defined by the EU standards and, in essence, their associated health benefits can be claimed for both SM and SMBP or foods containing them. In principle, SM and SMBP could be associated with various health claims (HC), including those related to energy-yielding metabolism, normal functioning of the immune system, and reduction of tiredness and fatigue.

## 1. Introduction

*Sobrassada de Mallorca* is made from finely minced pork mixed with salt, with a touch of pepper and the characteristic paprika. The latter is what gives it its red color, and the use of colorants is prohibited. It is also common to use authorized spices in variable proportions. The mixture is stuffed and left to cure for a few weeks or even months (depending on the stuffed size) so that a slow transformation occurs, in which part of the initial moisture is lost, and the food acquires its particular taste, texture, aroma, and flavors (according to the Consell Regulador Sobrassada de Mallorca PGI) [1,2]. Its origin dates back to the Roman Empire’s expansion of food preservation techniques throughout the Mediterranean Sea, including Mallorca and the other Balearic Islands, dealing with a primitive version of what would later develop into *Sobrassada* [3]. *Sobrassada de Mallorca* acquired its present form around the XVI century after the introduction of paprika as one of the main ingredients [4,5]. This fact became a fundamental milestone since paprika, apart from being a fundamental ingredient for the preservation of meat, gave the *Sobrassada* new organoleptic properties and its characteristic red color, in addition to added nutritional properties. The regulations on the specific denomination “*Sobrassada de Mallorca*” (SM)—including the ratio 30–60% and 70–40% of lean meat and fatty meat, respectively; the presence of paprika of the species *Capsicum annum* and/or *Capsicum longum*, between 4 and 7%; and salt content between 1.8–2.8%—are determined by the Regulatory Council of SM (*Consejo Regulador Sobrassada de Mallorca*, with a protected geographical indication (PGI) [6], and later, a new product was defined: the SM from black pig (SMBP). SM is prepared using pork from *Sus scrofa domestica*, and SMBP is prepared using pork from *Sus mediterraneus.* SMBP is made exclusively from the meat of the Mallorcan Black Pig, an indigenous pig breed native to Mallorca, associated with great cultural, gastronomic, and social significance for the island [3,7]. Even though the nutritional data on the general composition of *Sobrassada* is available in nutritional databases (598 kcal/100 gr, 61.4% of fats, 12.9% of proteins; 0% of carbohydrates), the differential composition of SM and SMBP controlled under PGI and their nutrient profiles have not been explored in detail.

Beyond the macronutrient composition, the micronutrient content is particularly relevant as it can be associated with beneficial health claims (HC) or effects [8]. The European Food Safety Authority (EFSA) has established a favorable opinion about the effect of some minerals and vitamins on health benefits. Most of them refer to the contribution of the ‘normal’ functions of the organism, and the Commission Regulation establishes the permitted HC related to each compound [6].

In this context, the present study aimed to analyze, characterize, and compare the nutritional composition of fifteen samples of SM and nine samples of SMBP and to assess possible properties and HC associated with some of their nutritional components.

## 2. Materials and Methods

### 2.1. Collection and Preparation of Samples

A total of 24 samples of *Sobrassada* were obtained from producers to ensure the inclusion of samples from all manufacturers of *Sobrassada* covered by the PGI between June and November 2021 (*n* = 15, SM) and between October and November 2022 (*n* = 9, SMBP). Samples were collected from processors once the food curing process was completed. The criteria to consider the curing process completed were pH < 4.5 and water activity <0.91. After collection, samples were kept up to a maximum of 4 months under temperature-controlled storage (6–7 °C) and protected from light until shipment for compositional analysis.

### 2.2. Determination of Nutritional Composition

All nutritional composition parameters, including the proximal, micronutrient, and FA composition, were analyzed by an independent laboratory accredited with International Organization for Standardization (ISO) standards, Eurofins Laboratory (Eurofins Scientific SE, Luxembourg City, Luxembourg) in October–November 2021 and December 2022 for SM and SMBP, respectively. Proximal composition (ash, moisture, energy, carbohydrates, protein, fats, sodium, and starch), vitamin composition, and mineral composition were analyzed by Eurofins Análisis Alimentario SLU (Madrid), Eurofins Vitamin Testing Denmark A/S., and Eurofins Ecosur (Murcia), respectively.

#### 2.2.1. General Parameters and Macronutrient Composition

Ash content was determined by incineration at 550 °C (Internal method of Eurofins, Test procedure: NU-TM3573), and moisture content was determined by gravimetry (Internal method of Eurofins, Test procedure: NU-TM3503). Carbohydrates were determined by calculation, starch was analyzed by a laboratory enzymatic internal method, and total sugars were analyzed using ion chromatography with pulsed amperometric detection (based on *Association of Official Analytical Chemists* (AOAC) 2000.17) [9]. Total fat content was analyzed through hydrolysis-extraction using the Soxhlet method [10]. Briefly, an extraction of the fat from the previously hydrolyzed and dried sample is performed using organic solvent (e.g., petroleum ether), followed by removal of the solvent, drying of the residue, and subsequent weighing after cooling (Internal method of Eurofins, Test procedure: NU-TM3504). Proteins were analyzed using the Dumas method (based on UNE-EN ISO 14891; ISO 16634-1) [11,12] (Table 1).

#### 2.2.2. Lipid Profile

The more common FAs in foods were included in the FA profile analysis. However, only the results of those that showed a substantial (≥1% total fat) mean concentration are further described. Additionally, results from alpha linolenic acid, dihydrolipoic acid, and lauric acid are presented since their dietary intake is correlated with a number of health effects. The complete list of FAs analyzed is as follows: Saturated: C8:0 (caprylic acid), C10:0 (capric acid), C12:0 (lauric acid), C13:0 (tridecanoic acid), C14:0 (myristic acid), C15:0 (pentadecanoic acid), C16:0 (palmitic acid), C17:0 (margaric acid), C18:0 (stearic acid), C20:0 (arachidic acid), and C22:0 (behenic acid). Monounsaturated: C14:1n5t (myristelaidic acid), C14:1n-5c (myristoleic acid), C15:1 n-5c (cis-10-pentadecenoic acid), C16:1 n7 cis (palmitoleic acid), C17:1 n7c (cis-10-Heptadecenoic acid), Trans C 18:1, C18:1 n12c (petroselinic acid), C18:1 n9c (oleic acid), C18:1n7c (cis-vaccenic acid), C20:1 n9c (gondoic acid), C22:1 n9c (erucic acid), and C24:1 n9c (nervonic acid). Polyunsaturated: Trans C18:2, C18:2 n6c (linoleic acid), C18:3 n6c (gamma-linolenic acid GLA), C18:3 n3c (alpha-linolenic acid ALA), C20:2 n6c (cis 11,14-Eicosadienoic acid), C20:3 n6c (dihomo-gamma-linolenic acid DHLA), C20:3 n3c (eicosatrienoic acid), C20:4 n6c (arachidonic acid), C22:2 n6c (13,16-Docosadienoic acid), C24:0 (lignoceric acid), C20:5n3c (eicosapentaenoic acid EPA), C22:5n 3c (docosapentaenoic acid DPA), and C22:6 n3c (Docosahexaenoic acid DHA).

FAs were determined by gas chromatography with flame ionization detection (GC-FID) (Internal method of Eurofins, Test procedure: CR-TM5707). Levels of MUFAs, polyunsaturated FAs (PUFAs), and saturated FAs (SFAs) were calculated by the sum of each type of FA.

#### 2.2.3. Micronutrient Composition

The content of minerals included in the analyses was determined by inductively coupled plasma mass spectrometry (ICP-MS), except sodium, which was measured by atomic absorption spectrometry (Internal method of Eurofins, test procedure: NU-TM3571).

Regarding vitamins, before the determination, vitamins were extracted from the sample as follows: Vitamin B_6_ was extracted in an autoclave by acid hydrolyzation, followed by an enzymatic treatment of dephosphorylation. Then, pyridoxamine was transformed into pyridoxal by reaction with glyoxylic acid in the presence of Fe^2+^ as a catalyst. Pyridoxal was then reduced to pyridoxine by sodium borohydride in an alkaline medium. Vitamin B_2_ was extracted from the sample by acid hydrolyzation and then by enzymatic dephosphorylation in an autoclave. Extraction of pantothenic acid from the sample was performed in an autoclave by a buffer. Vitamin B_12_ was extracted and subjected to cleaning using immunocolumns. Finally, vitamin B_3_ was extracted with hydrochloric acid at 100 °C. After that, vitamin B_2_, as defined in UNE-EN 14152:2014 [14], vitamin B_3_, as defined in UNE-EN 15652:2009 [15], and vitamin B_6_, as defined in UNE-EN 14164:2014 [17] were determined using high-performance liquid chromatography (HPLC). Specifically, vitamins B_6_ and B_2_ were determined with HPLC with fluorimetric detection (e.g., Ex: 468 nm; Em: 520 nm), vitamin B_3_ with RP-HPLC-FLD with post-column derivatization, and vitamin B_12_ was measured using HPLC with UV detection (based on J. AOAC 2008, vol 91 No 4 [18]). On the other hand, the extracted vitamin B_5_ was determined by measuring microbial growth (based on AOAC 945.74/45.2.05 (1990) [16]). Briefly, vitamin B_5_ was diluted in a culture medium that contained all the nutrients necessary for its development, *Lactobacillus plantarum* (ATTCC 8014) was inoculated, and its growth was measured by turbidimetry. The results were then compared with calibrated standards that allow for quantification of vitamin B_5_. Concerning the other vitamins, vitamin A (retinol form) was analyzed, as defined in UNE-EN 12823-1:2014 [13], using HPLC with a UV-diode array detector (DAD) at 325 nm; beta-carotene was determined as defined in EN 12823-2:2000 [19] using RP-HPLC on a C30 column and detected by a DAD at 450 nm; and vitamin E as defined in UNE-EN 12822:2014 [20] using HPLC with fluorometric detection (e.g., Ex: 290 nm, Em.: 527 nm). Additionally, to obtain the total vitamin A content, the following formula was applied: vitamin A = total retinol + 1/6 of β-carotene [21].

The analysis of ash, moisture, sodium, energy, proteins, carbohydrates, sugars, and FA profiles have been performed under the accreditation of UNE-EN ISO/IEC 17025:2017, ENAC 1094/LE2182 standard, complying with general requirements for the competence of testing and calibration laboratories. The analyses of the micronutrient composition were performed under the accreditation of UNE-EN ISO/IEC 17025:2017 ENAC 354/LE709 (minerals, except sodium) and DS EN ISO/IEC 17025 DANAK 581 (vitamins).

### 2.3. Nutrition and HC Assessment

Based on mineral and vitamin content, those present in *Sobrassada* in a significant amount [22] were selected to search the authorized HC linked to their significant content. HC were collected from the European Commission’s database of authorized claims [23].

### 2.4. Statistical Analysis

All analyses were performed using the statistics software SPSS v27.0 (IBM, Chicago, IL, USA). All data are expressed as the mean ± standard error of the mean (SEM). Single comparisons of compositional items between SM and SMBP samples were assessed by the Mann–Whitney U test. The 15% of the established NRV of each micronutrient was calculated to identify those micronutrients that SM and SMBP contain in significant amounts (considering values of at least 15% of NRV).

## 3. Results

### 3.1. General Parameters and Macronutrient Composition of SM and SMBP

SMBP showed a higher content of ash than SM, and both types of *Sobrassada* resulted in a similar level of moisture (Table 2). Additionally, both types of *Sobrassada* showed a minimal content of carbohydrates and sugars (<1%).

Both types of analyzed *Sobrassada* with PGI (SM and SMBP) showed around 578 kcal/100 g, with no differences between them. Results revealed the proportion of macronutrients was <0.5% of Kcal from carbohydrates, 89.4% from fat, and 7.91% from protein in SM, and <0.5% of Kcal from carbohydrates, 90.9% from fat, and 9.01% from protein in SMBP. Overall, the macronutrient content between both types of *Sobrassada* was similar.

### 3.2. FA Profile of SM and SMBP

The proportions of FAs in SM with PGI showed that more than 60% of total fat was unsaturated, and less than 40% was saturated. Specifically, each 100 g of SM resulted in 26.2 g of MUFAs and 9.99 g of PUFAs. The major MUFA of SM was oleic acid, and the major PUFAs were omega-6 fatty acids, especially linoleic acid. On the other hand, the analyses revealed that the major SFA was palmitic acid, which represented 63% of saturated fat (Table 3). SMBP showed a different FA profile; even though the unsaturated fat was also around 60% and the saturated fat was around 40%, the MUFA and SFA contents were higher than in SM, and the PUFA content was lower. Accordingly, palmitic acid, which was also the main SFA in SMBP, was higher in this type of *Sobrassada* compared to SM. In the same way, oleic acid and the other major MUFAs (vaccenic cis acid and palmitoleic acid) were higher in SMBP than in SM. Regarding PUFAs, SMPB had a lower content of linoleic acid, alpha linolenic acid, and dihydrolipoic acid compared to SM. The ratio of C18:2/C18:3 was lower in SMBP than in SM. It should be taken into account that in SMBP, the PUFAs were also mainly omega-6, as shown in SM (Table 3).

### 3.3. Micronutrient Composition (Minerals and Vitamins) of SM and SMBP

The minerals and vitamins that seemed to be present in a significant amount in *Sobrassada*, considering BEDCA data and their content in the food ingredients, were measured in SM and SMBP samples with PGI. Most of the analyzed minerals in SM and SMBP were present in a significant amount (at least 15% of the NRV per 100 g of product [22]). The analyses showed that SM contains a significant amount of iron, phosphorus, and selenium. Similarly, SMBP showed a significant content of phosphorus, selenium, and zinc, even though it showed lower levels of phosphorus compared to SM. Moreover, SMBP also revealed lower iron content, which was not a significant amount in SMBP, and higher levels of zinc compared to SM, which resulted in a significant amount only in SMBP (Table 4).

Vitamin content was similar in SM and SMBP, especially for vitamins A, B_2_, B_12_, and E. However, SMBP showed higher vitamin B_5_ and B_6_ content compared to SM; meanwhile, vitamin B_3_ was higher in SM. Despite these differences, both showed a significant amount of vitamins A, B_3_, B_6_, B_12_, and E (Table 4).

### 3.4. Potential Related HC Attributed to SM and SMBP

Foods that contain significant amounts of specific micronutrients are considered sources of them, and in accordance with the European Food Regulation, certain authorized HC can be attributed to them [24]. In this sense, as has been previously mentioned, the results showed that SM contained a significant amount of three minerals (iron, phosphorus, and selenium) and five vitamins (A, B_3_, B_6_, B_12_, and E) and could be associated with a total of 27 different health effects (Supplementary Table S1). On the other hand, the measured micronutrients that resulted in a significant amount in SMBP were phosphorus, selenium, and zinc, and the same five vitamins (A, B_3_, B_6_, B_12_, and E) observed in SM; thus, SMBP could be correlated to 35 different health effects (Supplementary Table S2). Among them, the contribution to the maintenance of immune function and the energy-yielding metabolism are the most representative health benefits related to the HC that may be associated with both types of *Sobrassada* (Supplementary Figure S1 and Supplementary Tables S1 and S2).

## 4. Discussion

*Sobrassada* is a traditional food from Mallorca that is enjoyed not only locally but also in other parts of Spain and around the world [25]. However, there are limited data available on its nutritional profile considering factors like the origin, production techniques, or the source of meat.

Data on the nutritional composition of the raw materials used in the production of *Sobrassada* [6,26], as well as of the *Sobrassada* (as generic food) or other similar meat-based products to *Sobrassada* (such as *chorizo*, which is a typical Spanish cured sausage) from BEDCA, suggest that *Sobrassada* could contain a set of micronutrients—possibly in a significative amount—defined by Regulation (EC) No 1169/2011 (defined as at least 15% of the nutrient reference values, NRV) [22]. Within this context, a total of 11 micronutrients were determined in different samples of SM and SMBP. These micronutrients include iron, phosphorus, zinc, selenium, riboflavin (vitamin B_2_), niacin (vitamin B_3_), pantothenic acid (vitamin B_5_), pyridoxine (vitamin B_6_), cyanocobalamin (vitamin B_12_), vitamin E (α-tocopherol), and vitamin A. Additionally, given the high proportion of fat present in *Sobrassada* according to data from the food composition database [26], there was a high interest in describing a detailed analysis of the lipid profile, particularly due to its relatively high content of monounsaturated FAs (MUFAs).

Briefly, the characterization of the macronutrient profile of *Sobrassada* with PGI performed in this study showed that the results are similar to the information provided by BEDCA [26] and by published data [1,25]. Specifically, analytical measurements on SM and SMBP showed a water content ranging between 13.0–30.0%, 50.3–68.4% of fat, and 9.6–13.8% of protein. Comparing SM and SMBP, the levels of fat stand out, which showed a tendency to be lower in SMBP, and the FA profile resulted in some differences when comparing SM to SMBP. Finally, the mineral and vitamin content of *Sobrassada* resulted in interest, as both types of *Sobrassada* contain a significant amount of minerals and vitamins (phosphorus and selenium, and vitamins A, B_3_, B_6_, B_12_, and E), which have been attributed health beneficial effects related to authorized HC by the European Commission.

The FA profile of SMBP has been previously studied by Gianelli et al. [1], showing a high content of MUFAs. Moreover, the authors revealed that PUFAs are oxidized during the curing process, which generates flavor compounds characteristic of its aroma [1]. The present data also revealed a high content of MUFAs (palmitoleic acid, vaccenic acid, and oleic acid) in SMBP, even more than in SM samples (54.2% versus 44.8% of total fat). Oleic acid stands out as the most abundant MUFA in both types of *Sobrassada*, and the consumption, in proper levels, of this FA has been related to beneficial effects in maintaining normal levels of LDL cholesterol [27], weight management [28,29], and other beneficial health effects [30,31,32,33]. Moreover, the present data showed that *Sobrassada* also notably contains PUFAs, with SMBP showing lower levels than MS (7.14% vs. 17.1% of fat); among them, linoleic acid is the most abundant PUFA in both types of *Sobrassada.* Linoleic acid consumption has been related to some beneficial effects, such as contributing to the maintenance of normal cholesterol levels [34], exhibiting anti-inflammatory properties [35,36], and others [37,38,39]. Furthermore, as has been reported by other authors [1], *Sobrassada* also contains a large proportion of SFAs (especially SMBP samples), for which excessive consumption, particularly palmitic acid, is associated with increased LDL circulating cholesterol, a risk factor for cardiovascular disease (CVD) [40,41,42,43]. Nevertheless, the *Sobrassada*’s SFA content and this particular FA are lower than in other pork-derived products, e.g., *chorizo* (28.91%) [44]. Interestingly, reducing dietary SFAs and replacing them with MUFAs and PUFAs has been linked to a reduced risk of CVD [40,45,46]; similarly for increasing the dietary intake of some unsaturated FAs such as linoleic (omega-6), α-linolenic (omega-3), and oleic (omega-9) acids (which are also present in *Sobrassada*) [27,47].

In addition to its remarkable lipid profile, the analysis of SM and SMBP showed a significant content of some vitamins and minerals. The notable content of those micronutrients is attributed to the ingredients used for *Sobrassada* production. Beyond pork, other ingredients contain some micronutrients. For instance, paprika, used for *Sobrassada* production [25], is rich in vitamins A, E, and C and other bioactive compounds (such as flavonoids, phenolic acids, and carotenoids), for which consumption has been associated with health benefits [4,48,49,50,51]. Apart from vitamins, iron, phosphorus, selenium, and zinc were selected to be measured in SM and SMBP. Of those, iron, phosphorus, and selenium were present in a significative content (at least 15% of NRV [22]) in MS, whereas phosphorus, selenium, and zinc were in SMBP. This is remarkable because the intake of these essential minerals, although in small quantities, is indispensable for the whole organism’s normal functioning [8], and nutritional studies indicate that their poor intake is associated with a higher risk of developing health issues [52,53]. It is crucial to emphasize the importance of meeting the optimal intake of these micronutrients to ensure normal bodily functions, including, for example, the immune system [8]. In fact, given that the link between the proper intake of some of these minerals and immune system maintenance is clear, the European Commission authorized HC for iron, selenium, and zinc, indicating that each one contributes to the normal function of the immune system [54,55,56]. This claim could be used only by those products that can be considered a source of these minerals [54,55,56], which means they must contain them in significant amounts [24], e.g., SM and SMBP. Moreover, there are other authorized HC related to the mineral content of SM and SMBP that are related to various beneficial health effects (Supplementary Tables S1 and S2, and Supplementary Figure S1). In addition to the mineral content, both types of *Sobrassada* contain a significant amount of vitamins A, B_3_, B_6_, B_12_, and E, for which deficiencies in dietary intake are associated with a higher risk of developing physiological disorders or pathological conditions [53]. For instance, optimal consumption of vitamins A, B_6_, and B_12_ is essential for the proper functioning of the components of the immune system, and suboptimal intake of these vitamins has been associated with an increased risk of contracting and experiencing more severe symptoms of COVID-19 [52,57]. In addition, regarding authorized HC by the European Commission, there are numerous claims that can be attributed to components of both types of *Sobrassada.* The most notable ones associated with different vitamins contained in *Sobrassada* are related to the normal function of the immune system (which is related to the source of vitamin A, vitamin B_6_, and vitamin B_12_ in SM and SMBP) [58,59,60]. However, according to Article 4 of the NHC Regulation, the EC should have already decided on limitations for HC on foods that do not qualify as nutritionally appropriate. This is especially relevant for *Sobrassada,* which contains a high amount of SFA, even though it should be taken into account that *Sobrassada* is rarely enjoyed on its own. The evolution of the native gastronomic culture has resulted in *Sobrassada* being a food always accompanied by others with properties that complement its nutritional profile. For example, the combination of *Sobrassada* with the traditional ‘*xeixa*’ bread, prepared with flour from Mallorca’s native wheat (*Triticum aestivum*), which has relatively low gluten and very low sodium content, results in a meal rich in fiber and other minerals.

It is essential to emphasize that the findings presented in this study refer to ‘*Sobrassada*’, both the SM and SMBP samples, prepared by manufacturers adhering to stringent criteria set forth by the Regulatory Council. These criteria encompass the use of carefully selected ingredients in specific proportions, as well as compliance with established standards for the breeding and feeding of black pigs in the case of SMBP. Consequently, the characteristics shown here can be exclusively ascribed ‘*Sobrassada de Mallorca*’ with a protected geographical indication. Therefore, the nutritional attributes outlined in this study may not be directly applicable to preparations from manufacturers outside the PGI standards or homemade preparations, which are traditional during winter months in Majorcan society. Variations in ingredients, proportions, and production methods could potentially lead to divergent nutritional profiles in such instances.

## 5. Conclusions

To the best of our knowledge, this is the first research where the nutritional composition of SM and SMBP is studied in detail, including their micronutrient content and their FA profiles. The results of this study show that *Sobrassada de Mallorca* is a fat-rich food, but it has an interesting FA profile that includes a higher proportion of unsaturated FA. Moreover, this traditional food also contains significant levels of specific micronutrients essential for the normal functioning of our bodily systems. Hence, both of the two main characteristic types of *Sobrassada* present relevant levels of vitamins A, B_3_, B_6_, B_12_, and E and the minerals phosphorus and selenium. Particularly, SM presents notable levels of zinc, while SMBP has an important content of iron. In principle, it is a food rich in essential micronutrients whose presence in significant quantities could be associated with more than 27 beneficial effects on health, according to the European regulatory framework.

## Figures and Tables

**Table 1:** Analytical methods used for the composition analysis ^1^.

Component	Method	Results Expressed as
Ash	Combustion 550 °C	g/100 g
Moisture	Gravimetry	g/100 g
Kcal	Internal method	kcal/100 g
Carbohydrates	Calculation	g/100 g
Starch	Internal enzymatic method	g/100 g
Sugars	IC-PAD	g/100 g
Fat	Soxhlet method	g/100 g
Fatty acids	GC-FID	% of total fat
Protein	Dumas method	g/100 g
Iron	ICP-MS	mg/100 g
Phosphorus	ICP-MS	mg/100 g
Selenium	ICP-MS	μg/100 g
Sodium	Atomic absorption spectrometry	mg/100 g
Zinc	ICP-MS	mg/100 g
Vit A (RE)	HPLC; EN 12823-1 2014 [13]	μg/100 g
Vit B_2_	HPLC; EN 14152:2014 mod. [14]	mg/100 g
Vit B_3_	RP-HPLC; EN 15652:2009 [15]	mg/100 g
Vit B_5_	Microbial growth; AOAC 945.74/45.2.05 (1990) [16]	mg/100 g
Vit B_6_	HPLC; EN 14164:2014 [17]	mg/100 g
Vit B_12_	HPLC with UV detection; J. AOAC 2008, vol 91 No 4 [18]	μg/100 g
B-carotene	HPLC; EN 12823-2:2000 mod. [19]	μg/100 g
Vit E	HPLC; EN 12822:2014. [20]	mg/100 g

^1^ Abbreviations: IC-PAD: ion chromatography with pulsed amperometric detection; GC-FID: gas chromatography with flame ionization detection; ICP-MS: inductively coupled plasma mass spectrometry; HPLC: high-performance liquid chromatography; UV: ultraviolet.

**Table 2:** Proximal composition of both *Sobrassadas*: SM and SMBP ^1^.

Component	SM	SMBP	*p*-Value
Ash (g/100 g)	3.33 ± 0.06	3.57 ± 0.08	0.037
Moisture (g/100 g)	21.4 ± 1.32	25.0 ± 1.70	0.115
Energy (kcal/100 g)	588 ± 13.5	561 ± 17.6	0.222
Carbohydrate (g/100 g)	<0.5 ± 0.09	<0.5 ± 0.06	-
Sugars (g/100 g)	<0.5 ± 0.07	<0.5 ± 0.06	-
Starch (g/100 g)	1.66 ± 1.09	<0.5 ± 0.00	-
Fat (g/100 g)	58.4 ± 1.61	56.7 ± 2.03	0.633
Protein (g/100 g)	11.6 ± 0.33	12.6 ± 0.34	0.060

^1^ The values of each nutritional parameter are expressed in grams per 100 g of product (g/100 g), except the values of total energy, which are expressed in kcal per 100 g of product (kcal/100 g). Results represent mean ± SEM (*n* = 15 for SM and 9 for SMBP). Statistical differences between SM samples vs. SMBP were assessed by Mann–Whitney U test, and the obtained *p*-values are shown. (-): statistics are not applied because the values are below the lower limit of quantification.

**Table 3:** Fatty acid characterization of both *Sobrassadas*: SM and SMBP ^1^.

Fatty Acid	SM	SMBP	*p*-Value
Mufas, total (%)	44.8 ± 1.45	54.2 ± 1.17	0.034
Fatty acid 16:1n7c palmitoleic acid (%)	2.16 ± 0.05	3.12 ± 0.12	<0.001
Fatty acid 18:1n7c vacenic cis acid (%)	2.79 ± 0.04	4.13 ± 0.20	0.020
Fatty acid 18:1n9c oleic acid (%)	39.7 ± 0.40	45.7 ± 0.87	<0.001
Pufas, total (%)	17.1 ± 0.51	7.14 ± 0.54	<0.001
Fatty acid 18:2n6c linoleic acid (%)	15.1 ± 0.47	6.11 ± 0.43	<0.001
Fatty acid 18:3n3c alpha linolenic acid (%)	0.95 ± 0.02	0.47 ± 0.07	<0.001
Fatty acid 20:3n6c dihydrolipoic acid (%)	0.14 ± 0.00	0.06 ± 0.03	0.001
Sfas, total (%)	36.6 ± 0.28	38.4 ± 0.72	0.016
Fatty acid 12:0 lauric acid (%)	0.09 ± 0.28	<0.05 ± 0.00	0.001
Fatty acid 14:0 miristic acid (%)	1.38 ± 0.03	1.40 ± 0.02	0.683
Fatty acid 16:0 palmitic acid (%)	23.0 ± 0.15	24.6 ± 0.28	<0.001
Fatty acid 18:0 stearic acid (%)	11.6 ± 0.16	11.9 ± 0.47	0.446
Omega-3 (%)	1.09 ± 0.04	0.54 ± 0.11	<0.001
Omega-6 (%)	16.0 ± 0.47	6.60 ± 0.44	<0.001
C18:2/C18:3 (ratio)	15.8 ± 0.16	14.2 ± 0.98	0.048

^1^ The mean values of total MUFAs, PUFAs, and SFAs are expressed as percentage of fat (%) ± Standard Error of the Mean (SEM) (*n* = 15 for SM and 9 for SMBP). Statistical differences between SM samples vs. SMBP were assessed by Mann–Whitney U test, and the obtained *p*-values are shown.

**Table 4:** Micronutrient composition of both *Sobrassadas*: SM and SMBP ^1^.

	SM	SMBP	*p*-Value
Minerals			
Iron (mg/100 g)	**2.53 ± 0.19**	1.91 ± 0.16	0.044
Phosphorus (mg/100 g)	**271 ± 17.1**	**186 ± 10.1**	0.002
Selenium (μg/100 g)	**17.3 ± 1.14**	**16.2 ± 1.30**	0.574
Zinc (mg/100 g)	1.05 ± 0.08	**1.69 ± 0.10**	<0.001
Sodium (mg/100 g)	1240 ± 0.07	1050 ± 0.06	0.083
Vitamins			
Vitamin A (total, μg/100 g)	**241 ± 12.2**	**232 ± 32.1**	0.859
Vitamin B_2_ (riboflavin, mg/100 g)	0.17 ± 0.01	0.19 ± 0.01	0.059
Vitamin B_3_ (niacin, mg/100 g)	**3.50 ± 0.12**	**2.43 ± 0.08**	<0.001
Vitamin B_5_ (pantothenic acid, mg/100 g)	0.64 ± 0.02	0.73 ± 0.04	0.035
Vitamin B_6_ (pyridoxine, mg/100 g)	**0.27 ± 0.01**	**0.35 ± 0.02**	0.004
Vitamin B_12_ (cyanocobalamin, μg/100 g)	**0.65 ± 0.03**	**0.56 ± 0.03**	0.091
Vitamin E (alpha-tocopherol, mg/100 g)	**2.81 ± 0.16**	**2.67 ± 0.29**	0.645

^1^ The mean values of each nutritional parameter are expressed correspondingly in milligrams per 100 g of product (mg/100 g) or micrograms per 100 g of product (μg/100 g) ± Standard Error of the Mean (SEM). Statistical differences between SM samples vs. SMBP were assessed by Mann–Whitney U test, and the obtained *p*-values are shown. The significant amount of each micronutrient is highlighted in bold (at least 15% of the NRV per 100 g of product [9]).

## Data Availability

The original contributions presented in the study are included in the article, further inquiries can be directed to the corresponding author.

## Supplementary Materials

The following supporting information can be downloaded at: https://www.mdpi.com/article/10.3390/foods13050761/s1, Figure S1: (a) number of health claims (HC) related to specific health benefits for SM; (b) number of HC related to specific health benefits for SMBP; Table S1: Health claims (HC) related to specific health effects for SM; Table S2: Health claims (HC) related to specific health effects for SMBP.
