# Supplementary material for: Biochemical Composition and Related Potential Nutritional and Health Properties of Sobrassada de Mallorca
Source: Foods. 2024 Feb 29;13(5):761. doi: 10.3390/foods13050761 (PMC10930987; doi:10.3390/foods13050761)

Supplementary Materials of “Biochemical composition and related potential nutritional and health properties of *Sobrassada de Mallorca*” by Galmés et al.

Supplementary Table S1. Health claims related to specific health effects for SM.

**Function of the immune system**	5
iron contributes to the normal function of the immune system	
selenium contributes to the normal function of the immune system	
vitamin A contributes to the normal function of the immune system	
vitamin B_6 contributes to the normal function of the immune system	
vitamin B_{12} contributes to the normal function of the immune system	
**Contribution to normal energy-yielding metabolism**	5
iron contributes to normal energy-yielding metabolism	
phosphorus contributes to normal energy-yielding metabolism	
niacin contributes to normal energy-yielding metabolism	
vitamin B_6 contributes to normal energy-yielding metabolism	
vitamin B_{12} contributes to normal energy-yielding metabolism	
**Reduction of tiredness and fatigue**	4
iron contributes to the reduction of tiredness and fatigue	
niacin contributes to the reduction of tiredness and fatigue	
vitamin B_6 contributes to the reduction of tiredness and fatigue	
vitamin B_{12} contributes to the reduction of tiredness and fatigue	
**Maintenance of normal skin and mucous membranes**	4
vitamin A contributes to the maintenance of normal mucous membranes	
vitamin A contributes to the maintenance of normal skin	
niacin contributes to the maintenance of normal mucous membranes	
niacin contributes to the maintenance of normal skin	
**Others**	32
iron contributes to normal cognitive function	
iron contributes to normal formation of red blood cells and hemoglobin	
iron contributes to normal oxygen transport in the body	
iron has a role in the process of cell division	
iron contributes to normal cognitive development of children	
phosphorus contributes to normal function of cell membranes	
phosphorus contributes to the maintenance of normal bones	
phosphorus contributes to the maintenance of normal teeth	
phosphorus is needed for the normal growth and development of bone in children	
selenium contributes to the protection of cells from oxidative stress	
selenium contributes to normal spermatogenesis	
selenium contributes to the maintenance of normal hair	
selenium contributes to the maintenance of normal nails	
selenium contributes to the normal thyroid function	
vitamin A contributes to normal iron metabolism	
vitamin A contributes to the maintenance of normal vision	
vitamin A has a role in the process of cell specialisation	

niacin contributes to normal functioning of the nervous system

niacin contributes to normal psychological function

vitamin B_6 contributes to normal cysteine synthesis

vitamin B_6 contributes to normal functioning of the nervous system

vitamin B_6 contributes to normal homocysteine metabolism

vitamin B_6 contributes to normal protein and glycogen metabolism

vitamin B_6 contributes to normal psychological function

vitamin B_6 contributes to normal red blood cell formation

vitamin B_6 contributes to the regulation of hormonal activity

vitamin B_{12} contributes to normal functioning of the nervous system

vitamin B_{12} contributes to normal homocysteine metabolism

vitamin B_{12} contributes to normal psychological function

vitamin B_{12} contributes to normal red blood cell formation

vitamin B_{12} has a role in the process of cell division

vitamin E contributes to the protection of cells from oxidative stress

Supplementary Table S2. Health claims (HC) related to specific health effects for SMBP.

**Function of the immune system**	5
selenium contributes to the normal function of the immune system	
zinc contributes to the normal function of the immune system	
vitamin A contributes to the normal function of the immune system	
vitamin B_6 contributes to the normal function of the immune system	
vitamin B_{12} contributes to the normal function of the immune system	
**Contribution to normal energy-yielding metabolism**	4
phosphorus contributes to normal energy-yielding metabolism	
niacin contributes to normal energy-yielding metabolism	
vitamin B_6 contributes to normal energy-yielding metabolism	
vitamin B_{12} contributes to normal energy-yielding metabolism	
**Maintenance of normal skin and mucous membranes**	4
vitamin A contributes to the maintenance of normal mucous membranes	
vitamin A contributes to the maintenance of normal skin	
niacin contributes to the maintenance of normal mucous membranes	
niacin contributes to the maintenance of normal skin	
**Reduction of tiredness and fatigue**	3
niacin contributes to the reduction of tiredness and fatigue	
vitamin B_6 contributes to the reduction of tiredness and fatigue	
vitamin B_{12} contributes to the reduction of tiredness and fatigue	
**Protection of DNA, proteins and lipids from oxidative damage**	3
selenium contributes to the protection of cells from oxidative stress	
zinc contributes to the protection of cells from oxidative stress	
vitamin E contributes to the protection of cells from oxidative stress	
**Others**	41
phosphorus contributes to normal function of cell membranes	
phosphorus contributes to the maintenance of normal bones	
phosphorus contributes to the maintenance of normal teeth	
phosphorus is needed for the normal growth and development of bone in children	
selenium contributes to normal spermatogenesis	
selenium contributes to the maintenance of normal hair	
selenium contributes to the maintenance of normal nails	
selenium contributes to the normal thyroid function	
zinc contributes to normal carbohydrate metabolism	
zinc contributes to normal cognitive function	
zinc contributes to normal DNA synthesis	
zinc contributes to normal fertility and reproduction	
zinc contributes to normal macronutrient metabolism	
zinc contributes to normal metabolism of fatty acids	
zinc contributes to the maintenance of normal bones	
zinc contributes to the maintenance of normal hair	
zinc contributes to the maintenance of normal nails	
zinc contributes to the maintenance of normal skin	
zinc contributes to the maintenance of normal vision	
zinc has a role in the process of cell division	

zinc contributes to normal acid-base metabolism
zinc contributes to normal metabolism of vitamin A
zinc contributes to normal protein synthesis
zinc contributes to the maintenance of normal testosterone levels in the blood
vitamin A contributes to normal iron metabolism
vitamin A contributes to the maintenance of normal vision
vitamin A has a role in the process of cell specialization
niacin contributes to normal functioning of the nervous system
niacin contributes to normal psychological function
vitamin B_6 contributes to normal cysteine synthesis
vitamin B_6 contributes to normal functioning of the nervous system
vitamin B_6 contributes to normal homocysteine metabolism
vitamin B_6 contributes to normal protein and glycogen metabolism
vitamin B_6 contributes to normal psychological function
vitamin B_6 contributes to normal red blood cell formation
vitamin B_6 contributes to the regulation of hormonal activity
vitamin B_{12} contributes to normal functioning of the nervous system
vitamin B_{12} contributes to normal homocysteine metabolism
vitamin B_{12} contributes to normal psychological function
vitamin B_{12} contributes to normal red blood cell formation
vitamin B_{12} has a role in the process of cell division

Supplementary Figure S1. (**A**) Number of health claims (HC) related to specific health benefits for SM; (**B**) Number of health claims related to specific health benefits for SMBP.

A)

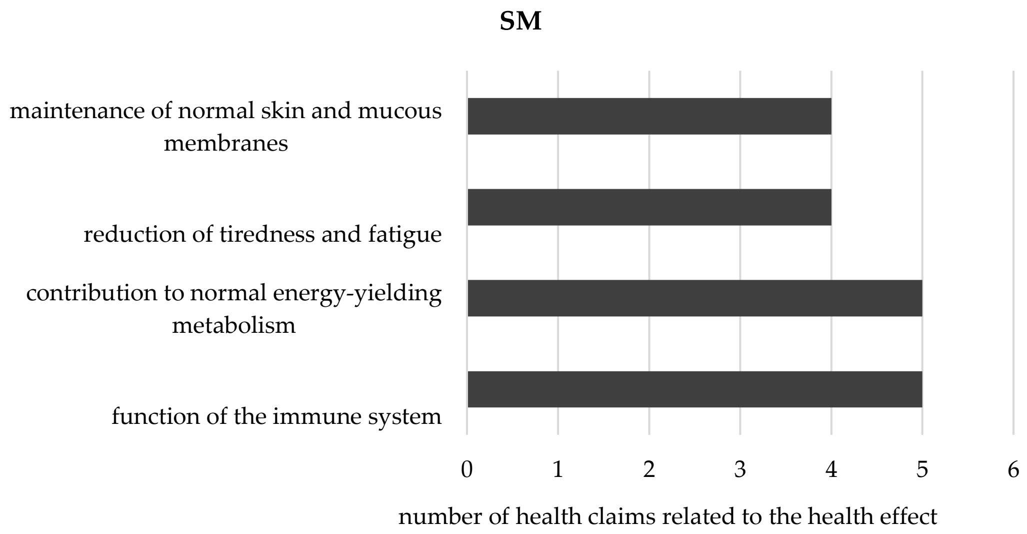

B)

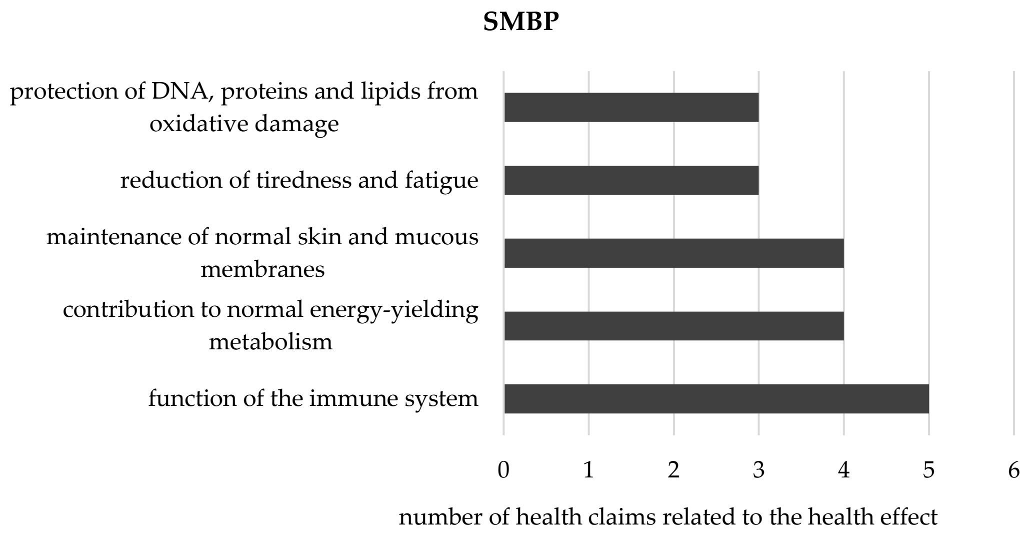

Supplement: Supplementary file 1 [file foods-13-00761-s001.zip › foods-2842702-supplementary.pdf]
